# Low Level Laser Therapy for the Treatment of Diabetic Foot Ulcers: A Critical Survey

**DOI:** 10.1155/2014/626127

**Published:** 2014-03-16

**Authors:** Kathrin H. Beckmann, Gesa Meyer-Hamme, Sven Schröder

**Affiliations:** HanseMerkur Center for Traditional Chinese Medicine, University Medical Center Hamburg-Eppendorf, Martinistraße 52, 20246 Hamburg, Germany

## Abstract

Diabetic foot ulcers as one of the most common complications of diabetes mellitus are defined as nonhealing or long-lasting chronic skin ulcers in diabetic patients. Multidisciplinary care for the diabetic foot is common, but treatment results are often unsatisfactory. Low level laser therapy (LLLT) on wound areas as well as on acupuncture points, as a noninvasive, pain-free method with minor side effects, has been considered as a possible treatment option for the diabetic foot syndrome. A systematic literature review identified 1764 articles on this topic. Finally, we adopted 22 eligible references; 8 of them were cell studies, 6 were animal studies, and 8 were clinical trials. Cell studies and animal studies gave evidence of cellular migration, viability, and proliferation of fibroblast cells, quicker reepithelization and reformed connective tissue, enhancement of microcirculation, and anti-inflammatory effects by inhibition of prostaglandine, interleukin, and cytokine as well as direct antibacterial effects by induction of reactive oxygen species (ROS). The transferral of these data into clinical medicine is under debate. The majority of clinical studies show a potential benefit of LLLT in wound healing of diabetic ulcers. But there are a lot of aspects in these studies limiting final evidence about the actual output of this kind of treatment method. In summary, all studies give enough evidence to continue research on laser therapy for diabetic ulcers, but clinical trials using human models do not provide sufficient evidence to establish the usefulness of LLLT as an effective tool in wound care regimes at present. Further well designed research trials are required to determine the true value of LLLT in routine wound care.

## 1. Introduction

Diabetes mellitus is one of the most common diseases worldwide. The prevalence of diabetes worldwide is estimated to be more than 371 million people and the number of people with diabetes is increasing in every country [1, 2]. One of the most common complications of diabetes mellitus is the diabetic foot syndrome [3]. It is defined as nonhealing or long-lasting chronic skin ulcers in diabetic patients. The diabetic foot syndrome is one of the most prevalent causes of nontraumatic limb amputations. Diabetic foot problems have a significant financial impact on the national health system and on patients' quality of life [4].

### 1.1. Risk Factors

A diabetic foot syndrome is a result of multifactorial occurrences due to different causes like peripheral neuropathy (sensory, motoric, and autonomic), peripheral arterial occlusive disease, or others, for example, limited joint mobility, foot deformation, and improper footwear.

### 1.2. Classification of Diabetic Foot Ulcers

Diabetic ulcers can be classified on the basis of severity as “mild (superficial and limited in size and depth), moderate (deeper or more extensive), or severe (accompanied by systemic signs or metabolic perturbations)” or in grades using the Wagner and Armstrong ulcer grade classification [5, 6]. Wagner grade 0–5 divides ulcers from a pre- or postulcerative lesion up to gangrene of the foot. Armstrong A–D adds the (non) existence of infection, ischemia, or both together. A compilation of the Wagner and the Armstrong ulcer classification system is shown in Table 1.

### 1.3. Conventional Treatment Methods

Therapeutic methods include different kinds of wound cleaning, debridement, skin grafting, antibiotics, vasodilators, pain management, and different types of bandages up to use of fly maggots [7]. But even in already existing multidisciplinary care systems for diabetic foot ulcers, treatment is still difficult and treatment results are often unsatisfactory.

### 1.4. Low Level Laser Therapy as a New Treatment Method

Low level laser therapy (LLLT), also called soft laser, is known to supply direct biostimulative light energy to body cells. The absorbed laser energy stimulates molecules and atoms of cells but does not cause rapid or significant increase in tissue temperature [8].

While most LLLT devices illuminate the treatment area from a certain distance, the term LLLT describes also a new method of laser acupuncture, called laser needle. Laser needles are not needles and not inserted into the skin; optical light fibers are fixed on the acupuncture points in an upright position. Thus, a high optical density can be achieved at the end of the fiber, minimizing light scattering that occurs at the surface of the skin. The therapeutic effects are of similar dimension to those evoked by manual needle acupuncture [9].

Different laser wavelengths have different depths of penetration into human tissue. Red laser has a deeper penetration depth than violet, blue, green, or yellow. Infrared and near infrared light are not visible, but it have been demonstrated to penetrate human tissue deeper than visible red light [10].

Bichromatic laser needles combine wavelengths, for example, of red (685 nm) and infrared (785 nm) light. Their penetration depth in human skin is 2-3 cm. Patients usually do not feel the beginning of the treatment, but 5–10 minutes later many patients report a pleasant warm and sometimes vibrating feeling in some treated areas.

Blue light (405 nm) is supposed to have a bactericidal effect on the tissue surface.

Other technical parameters are output power, power density, energy density, dose range, and continuous or pulsed laser [10].

Low-energy laser radiation was found to have a stimulating effect on cells, and high-energy radiation had an inhibiting effect. The application of lasers to stimulate wound healing in cases of nonhealing ulcers has been recommended [11]. In healthy volunteers, a randomized, triple-blind, placebo-controlled trial was performed. Twenty-two healthy humans got two standardized abrasions on the anterior forearm and afterwards a treatment with LLLT (with a 46-diode cluster head: 660 nm–820 nm) or a sham 46-diode cluster head. LLLT resulted in enhanced healing as measured by wound contraction. In contrast to the sham group, in the laser group, not only treated wounds became smaller but also untreated wounds (on the same arm). Hopkins et al. reasoned that LLLT may furthermore produce an indirect healing effect on surrounding tissues [12].

## 2. Methods

A systematic review of relevant literature was done by database research. Literature searches were conducted in the following databases from their inception through January 2013: MEDLINE, PubMed, BIOSIS, Embase, Cochrane Database, Thieme, Springer, Kluwer, and China National Knowledge Infrastructure (CNKI). The following MeSh terms were used individually or combined in appropriate language forms (Chinese, English, and German): diabetic foot ulcer, diabetic foot syndrome, DFS, chronic wound, diabetic wound, ulcus cruris, diabetic ulcus cruris, chronic ulcers in foot in diabetes, cutaneous wound, diabetic foot infection DFI, Traditional Chinese Medicine, Chinese Medicine, acupuncture, moxibustion, laser, and low level laser (LLLT). We identified 1764 articles. Articles concerning other than diabetic foot ulcer and articles concerning treatment methods other than acupuncture or irradiation were excluded.* In vivo* and* in vitro* studies and human and animal experimental studies were included. Finally, we adopted 22 eligible references, 8 of them were cell studies, 6 were animal studies, and 8 were clinical studies.

## 3. Results

### 3.1. Cell Studies

There is not yet a unique explanation about the biometrical and histological modes of functioning of laser therapy in the treatment of diabetic ulcers. But in the literature, various fundamental research studies trying to analyze several effects of LLLT on tissue repair mechanisms can already be found.

Cell studies with cultured human keratinocytes, endothelial cells, and fibroblasts indicated potential effects of near-infrared light in the treatment of chronic skin ulcers. After irradiation of the cells, the production of transforming growth factor (TGF)-*β*1 and matrix metalloproteinase (MMP)-2 was examined by enzyme immunoassay, zymography, and reverse transcription polymerase chain reaction (PCR). A biostimulatory effect of near-infrared irradiation was shown by a significant elevation of TGF-*β*1 and MMP-2 content in the medium of cultured cells. Irradiated fibroblasts also showed an upregulated amount of MMP-2 mRNA. These results suggest that near-infrared irradiation may accelerate wound closure [21, 22].

Houreld and Abrahamse tested the positive effect of low-intensity laser irradiation (LILI) of different wavelengths on cellular migration, viability, and proliferation in diabetic wounded and unwounded human skin fibroblast cells. They compared cellular morphology and migration (determined microscopically), cellular viability (determined by adenosine triphosphate (ATP) luminescence), and proliferation (determined by basic fibroblast growth factor expression and alkaline phosphatase activity). While diabetic wounded cells irradiated at 1,064 nm showed a lesser degree of migration, viability, and proliferation, cells irradiated at 632,8 nm showed a higher degree of haptotaxis and migration as well as ATP luminescence compared to cells irradiated at 830 nm [21]. In conclusion, diabetic wounded cells have more benefit in wound healing from irradiation in the visible range (632, 82 nm) than from the infrared range [23].

Few experimental studies of laser irradiation of human and animal cells in culture document positive photobiomodulatory effects of laser irradiation. Various types of cells involved in wound or soft-tissue repair or cell lines relating to soft tissues (human and animal stem cells, endothelial cells, smooth muscle cells, keratinocytes, fibroblasts, and others) respond differently to irradiation, also depending on irradiation parameters. [21, 22, 24–28] LLL irradiation resulted in an increased fibroblast proliferation* in vitro* [28]. Laser irradiation can promote cell migration and cell proliferation by stimulating mitochondrial activity and maintaining viability without causing damage to the wounded cells [24].

Additionally, the kinetics of reactive oxygen species (ROS) generation is influenced by laser irradiation and found to depend strongly on the laser fluence and not on the laser intensity [27].

### 3.2. Animal Studies

Diverse animal experiments indicate effects on the wound healing process [29–34].

In an excision model in rats (nondiabetic), not only red light (630 nm) but also blue light (470 nm) from light-emitting diode (LED) lamps improved perfusion by release of nitric oxide from nitrosyl complexes with haemoglobin, enhanced epithelialization, and elevated keratin-10 mRNA level. Recovery of mitochondria inhibited by nitric oxide (NO) gas was alleviated by blue light through the release of NO from mitochondrial complexes. NO induces endothelial cell migration by activating growth factors. In conclusion, blue light might improve wound healing via the NO pathway. One week after wound excision, the wound area was 50% smaller (*P* < 0,05) in the blue light group compared to the not illuminated control. Blue light especially enhances epithelialization even to a greater extent than red light does. Concerning the depth of granulation tissue, there was no significant influence either for red or for blue light [29].

Positive effects of gallium-aluminium-arsenide (GaAlAs)-laser, gallium-arsenide (GaAs) laser, and Dersani (linoleic acid) healing ointment on skin wounds in Wistar rats were determined in a study of Gonçalves et al. [30]. After irradiation, lesions were analyzed and the tissues were studied by electron microscopy, histology, and immunohistochemistry. Significant results for the wound closing rate were obtained for the treatment group with GaAs laser 4 J/cm^2^. In the treatment group with GaAlAs-laser, 30 J/cm^2^, the highest concentration of type III collagen fibers was found. For stimulating the production of type I and type III collagens, an energy density of 30 J/cm^2^ was most efficient. In terms of the synthesis of type I collagen, and mainly in speeding up the rate of wound closing, the use of 4 J/cm^2^ was more effective. In conclusion, laser therapy reduced the inflammatory reaction, induced increased collagen deposition, and stimulated a greater proliferation of myofibroblasts in experimental cutaneous wounds [29, 35].

Even a single low level laser (830 nm near-infrared, 1,3 J/cm^2^) treatment accelerated cutaneous wound healing in a rat model. Biometrical and histological analysis indicated faster lesion contraction showing quicker reepithelization and reformed connective tissue with more organized collagen fibers in irradiated wounds [31].

### 3.3. Randomized Clinical Trials

Randomized clinical trials correlate cellular effects and biologic processes and determine the utility of LLLT in cutaneous wound healing [35]. In a double-blind randomized placebo- controlled study, the healing effect of combined 660 and 890 nm LED laser treatment on twenty-three diabetic leg ulcers was tested by Minatel et al. Mean ulcer granulation and healing rates were significantly higher in the treatment group than in the placebo group at each of 15, 30, 45, 60, 75, and 90 days of treatment. Placebo-treated ulcers were cleaned, dressed with 1% silver sulfadiazine cream, and treated with placebo laser radiation < 1.0 J/cm^2^. During the initial 30 days, they even worsened. Ulcers in the treatment group got the same treatment but a 3 J/cm^2^ dose. At day 30, ulcers in the treatment group had achieved 56% more granulation and 79,2% faster healing than the placebo group and similarly higher rates of granulation and healing were maintained throughout. In the treatment group, 58,3% of ulcers had healed fully and 75% had achieved 90–100% healing by day 90. In contrast, in the placebo group, only one ulcer healed fully and no ulcer attained more than 90% healing [17].

In a randomized clinical trial, Zhou et al. explored the healing of irradiated (633 nm) chronic foot ulcers in 60 patients. 28 patients got conventional therapy and 32 received conventional therapy plus LLLP. There were 14 diabetic patients in the conventional treatment group and 18 diabetic patients in the conventional treatment plus LLLP group. (The other causes of ulcers were not described in detail.) Ulcers were evaluated by size reduction and immunohistochemical analysis of heat shock protein 70 (HSP70) positive cells. Protein and mRNA expressions of heat shock factor 1 (HSF1) and HSP70 were determined by reverse transcription polymerase chain reaction (RT-PCR) and Western blotting. Compared to the traditional therapy group and to the normal skin sections, as a control group, the expression of HSF1 and HSP70 in the laser group was significantly higher, as observed on the gray scale of the Western blot bands, just like the RNA levels of HSF1 and HSP70 by RT-PCR. Due to the mechanism of laser-activated endogenous heat shock protection in cells in wound surfaces, LLLT plays a facilitating role in the healing process of chronic dermal ulcers [16].

Schindl et al. found in a randomized, double-blind, placebo-controlled study, an increase of temperature after a single treatment with low-intensity laser irradiation (632,8 nm) as a sign of improved circulation in the skin of patients with diabetic microangiopathy [13]. In another study, Schindl et al. examined 20 patients with different causes of ulcers (diabetes (*n* = 8), arterial insufficiency (*n* = 5), radio damage (*n* = 4), and autoimmune vasculitis (*n* = 3)) and compared the number of necessary treatments until full wound closure. Ulcers due to radio damage healed significantly faster than those caused by diabetes. Wound healing in autoimmune vasculitis required longer time than in radiodermatitis, although the difference was not significant. Wound size was found to be an important factor for the healing time, whereas duration of previous conventional treatment and wound depth showed no effect [14]. Kaviani et al. treated twenty-three patients with a diabetic foot wound for at least three months additionally to conventional therapy either with a placebo treatment (*n* = 10) or LLLT (*n* = 13). It was a double-blind randomized clinical trial. A laser with wavelength of 685 nm and energy density of 10 J/cm^2^ was used. Comparison of ulcer size reduction and complete healing showed that LLLT can accelerate the healing process of chronic diabetic foot ulcers, hence shortening the period of complete healing. In the LLLT group, the size of ulcers decreased significantly at week four. In LLLT group, eight patients had complete healing after 20 weeks and in the placebo group only three patients experienced complete wound healing. Though the difference was not statistically significant, the mean time of complete healing in LLLT patients (11 weeks) was less than that in placebo patients (14 weeks) [18].

Saltmarche et al. tested the effectiveness of low level laser therapy (785 nm) for wound healing combined with the Extendicare Wound Prevention and Management Program at a Canadian Extendicare nursing area. They used infrared laser clusters of 16 × 5 mW and a 50 mW source of both 785 nm and applied 2 to 4 joules at each site, dependent on the pigment coating of the skin. The treatment was affected daily for 5 days in the first week and 3 times weekly from the 2nd to the 9th week or until ulcer healing. Twenty-one open wounds and four “at risk” closed areas were treated. 61,9% of the chronic (> or = 3 months duration) and acute (<3 months duration) ulcers due to pressure, venous insufficiency, and diabetes were included. In the first week, wounds were treated five times per week, thereafter three times a week for eight more weeks. 61,9% of the open wounds achieved significant improvement, measured as the size of the wound area. 42,8% had 100% closure. 14,3% had some improvement; 23,8% showed no change. There was no significant difference between chronic and acute wounds. No negative effects of the laser therapy were encountered [15].

Landau et al. investigated the effect of broadband visible light (400–800 nm) in a double-blind, placebo-controlled, randomized study on 16 patients suffering from diabetic or venous foot ulcers. The treatment group (*n* = 10) received wound illumination twice daily with 43.2 J/cm² while the placebo group (*n* = 6) received wound illumination in the same device with only 2.4 J/cm² which was declared as nontherapeutic. All patients received conventional wound care. At the end of the follow up, all of the wounds in 9 of the treatment group patients were closed (90%) whereas in the placebo group, only 2 of 6 patients (33%) had closed wounds, judged by Wagner's classification for foot ulcer and wide/length measurement. There were no adverse therapy effects [20].

The latest study of Kajagar et al. compared diabetic ulcer healing dynamics in 68 patients. They were randomized into a LLLT plus conventional care group which was compared with conventional care alone. On the basis of the ulcer size, the duration of exposure was calculated to deliver 2–4 J/cm_2_ at 60 mW, 5 kHz,daily for 15 days. The ulcer floor and edge were irradiated. There was a significant percentage of ulcer reduction in the LLLT group compared with conventional care alone [19].

A summary of the clinical trials is shown in Table 2.

## 4. Discussion

Diabetic foot syndrome as a chronic complication of Diabetes mellitus is a major therapeutic challenge [36]. It has a high financial impact and a severe effect on the patients' quality of life and can even lead to limb amputation [4]. Since diabetic foot ulcers are often difficult or impossible to treat with actual standard treatment methods [37], the search for further treatment options is necessary.

LLLT, as a noninvasive, pain-free method with minor side effects, has been considered as a possible treatment option for the diabetic foot syndrome. There is not yet a unique explanation of the mode of functioning of laser therapy in the treatment of diabetic ulcers. But in the literature, there are various studies trying to analyze several effects of LLLT on tissue repair mechanisms: cell and animal studies suggest a promotion of wound healing by laser irradiation due to improvement of different factors* in vitro* (playing an essential role in various tissue repair mechanisms) [16, 21, 22, 24, 26–28, 38].

Biometrical and histological analysis indicated “faster lesion contraction showing quicker reepithelization and reformed connective tissue with more organized collagen fibers” in irradiated wounds [32]. Laser therapy reduces the inflammatory reaction and provokes a greater proliferation of myofibroblasts in experimental cutaneous wounds [31, 38].

Stimulation of cell division and cell growth of fibroblasts plays an important role in wound healing [23]. Different wavelengths of low-intensity laser irradiation (LILI) have been tested on cellular migration, viability, and proliferation in diabetic wounded and unwounded human skin fibroblast cells. Cells irradiated at 632,8 nm showed a higher degree of haptotaxis and migration as well as ATP luminescence as compared to cells irradiated at 830 nm. These results may lead to the conclusion that diabetic wounded cells have more benefit in wound healing from irradiation in the visible range than in the infrared range [21]. However, since near-infrared light has a deeper penetration rate than visible red light, deeper ulcers* in vivo* might require the use of near infrared laser therapy [11].

Red light laser (630 nm) as well as blue light Laser (470 nm) can improve perfusion by release of nitric oxide from nitrosyl complexes with haemoglobin, enhanced epithelialization, and elevated keratin-10 mRNA level. Blue light also facilitated the recovery of mitochondria inhibited by NO gas by release of NO from mitochondrial complexes, so an improved wound healing via the NO pathway induces endothelial cell migration by activating growth factors, resulting in an increase keratin expression [29].

Anti-inflammatory effects of laser therapy can be explained by inhibition of prostaglandine, interleukin [39], and cytokine [40] in cell and animal models.

*In vitro* experiments with a low-power laser (415 nm) showed a direct antibacterial effect on* S. aureus* and* E. coli* by induction of ROS. [41, 42] LLLT also can increase the diameter and blood flow velocity of the peripheral arterioles and can enhance the microcirculation [23].

The transferral of information from* in vitro* studies or animal models is not always directly possible, so it is of high interest to test these methods in humans. There are a limited number of reviews on LLLT for wound healing. Most of them are presenting data on wound healing in general. Lucas et al. (2000) wrote a review on the effects of LLLT on wound healing. They concluded that there are no scientific arguments for routine application of low level (infrared) laser therapy on wound healing in patients with decubitus ulcers, venous leg ulcers, or other chronic wounds, and knowledge of wound care can only be improved by additional evidence from further clinical research [43]. In 2005, Posten et al. again criticized other studies assessing the qualitative and quantitative sufficiency of evidence for the efficacy of LLLT in promoting wound healing. Studies did not sufficiently assess the mechanism, whether photodermal, photochemical, or photomechanical, whereby LLLT may be exerting its effect. Posten et al. emphasize: “To better understand the utility of LLLT in cutaneous wound healing, good clinical studies that correlate cellular effects and biologic processes are needed” [35]. At that state, the majority of studies about the use of LLLT in human wounds did not demonstrate any benefit.

Another review with meta-analysis of 24 studies from 2004 concluded that LLLT is an effective tool for promoting wound repair, but they looked on studies on different wounds like bed sores, venous ulcers, diabetic ulcers, or surgical wounds [44].

A review from 2008 by Sobabanko and Alster discussed 12 randomized controlled trials of chronic cutaneous ulceration with a focus on venous leg ulcers and decubitus ulcers. They concluded that LLLT in humans does not improve wound healing and advised that after research, focused on cellular and molecular mechanisms of LLLT, larger and better controlled studies in humans must be performed to determine the appropriate laser parameters and treatment protocol [45].

Another review with meta-analysis from 2011 by Bjordal et al. focused on the treatment of cancer therapy-induced oral mucositis with LLLT. They concluded that the material was consistently in favour of LLLT in both in the prevention of oral mucositis occurrences and reductions of severity, pain, and duration of oral mucositis ulcers [46].

There is one recent review by Kwan et al. focusing on diabetic foot ulcer, but trials on all kinds of electrophysical therapies including LLLT were evaluated [47]. They included only two papers dealing with LLLT for the diabetic foot ulcer by Minatel et al. [17] and Kaviani et al. [18], which are part of our review as well.

While the effect of LLLT has shown to be different in certain etiologies of ulcers [14], a sophisticated evaluation of the properties of LLLT in different diseases is mandatory.

This is of particular relevance, because, for example, the majority of studies of LLLT for the treatment of venous ulcers showed no significant effect [15, 48–50] while a minority of studies delivered positive results [51–53].

In contrary, the results of the identified paper in this review showed positive results for the treatment of diabetic foot ulcers by LLLT. To our knowledge, our paper is the first review in English on LLLT for diabetic foot ulcers; and more studies are included than in prior reviews. However, only 8 trials deal with diabetic foot ulcers and three of these studies mix or compare diabetic etiology [14–16] with other causes of foot ulcers like decubitus ulcers and venous ulcers. So, the limited number and the heterogeneity of the measured parameters do not allow a meta-analysis of this topic.

Only very few statements about the cost-benefit ratio of laser therapy in the treatment of diabetic ulcers compared to standard treatment could be found in the current literature. In one study, low level laser treatment is evaluated as “easy to learn and use, effective for the majority of their residents, worth the additional time”. Saving of costs for eight wounds was described as 117,50 US-$ per month in addition to savings in nursing time amounting to 22.5 hours [15].

The diabetic ulcer as a widespread and common complication of diabetes mellitus is unfortunately often unsatisfactory curable using the actual standard treatment methods. In addition to LLLT, there already exist a number of other therapeutic approaches: different types of wound debridement, use of antimicrobials, use of dressings in wounds, topical negative pressure, hyperbaric oxygen treatment, electrical, electromagnetic, shockwave and ultrasound therapies, growth and cell biology factors, cell products and tissue engineering, bioengineered skin and skin grafts, and adjuvant therapies. In a critical review about these different treatment options, Gottrup and Apelqvist concluded that almost all studies referring to this topic are of too low quality to issue a significant statement about their benefit in the treatment of diabetic ulcers. “There is a substantial number of emerging technologies of potential value in the treatment of complex wounds, but there is an urgent need to increase the quality of clinical studies” [37].

As shown in this review, the quality of studies is still a problem. Subsuming the quintessence of the available study data by now, the majority of data show a potential benefit of low level laser therapy in wound healing of diabetic ulcers. But there are a lot of different aspects in the studies limiting final evidence about the actual output of this kind of treatment method. The number of trials is low and shows low quality and different methodical failures. Studies lack information about implementation and registration, methods of blinding, participant flow, and recruitment analysis. In most studies, the sample size is too low for further analysis and significant results [16–18, 52].

Sometimes methodological procedure or the type of laser used [16], details of other interventions [51], or practitioner background are explained inadequately. Often, control groups exist in form of different kinds of treatment methods like debridement, just cleaning the wound, Chinese herbal ointments or dressings, or irradiation of different wavelength but no nontreatment control groups.

However, nontreatment of chronic diabetic ulcer has to be considered as unethical, so at least a standard podiatric treatment is necessary in all study groups, and LLLT could be tested in combination with these types of treatments.

The methods of treatment varied a lot: different wavelengths, combined wavelengths, frequency and duration of irradiation, and combination of various treatment methods [16, 51, 52, 54].

Outcome measurement differed as well and some types were more subjective than objective: pain scales, subjective scales, and size of wounds determined by pictures, consequently, resulting in a lack of adequate statistical comparison [12, 54].

Some studies included not only diabetic ulcers but also ulcers due to venous insufficiency, peripheral artery occlusive disease, and others [16].

It is conceivable that only rarely adverse effects occurred; most studies did not even mentioned whether adverse effects were observed or not.

In summary cell studies, animal studies and clinical studies give enough evidence to continue research on LLLT for diabetic ulcers, but clinical trials using human models do not provide sufficient evidence to establish the usefulness of laser therapy as a standard tool in wound care regimes at this state. Further, well-designed research trials are required to determine the true value of laser therapy in routine wound care [55].

If researchers do not want their results to be only anecdotal, they have to take international quality standards for clinical trials into account. These are available for effective research on the treatment of chronic wounds. Incorporation of these recommendations in future study designs is highly desirable and would substantively advance the quality of wound care studies [55] and might elude the role of laser therapy in the treatment of diabetic ulcers.

## 5. Conclusion

Since current therapies are variable in their ability to induce complete healing, there remains a need to develop adjunctive treatments that can improve or accelerate the healing process in diabetic foot ulcers. The available studies about LLLT as treatment methods of diabetic ulcers give positive results and encourage further investigations. In order to obtain conclusive evidence of low level laser in treating diabetic foot ulcers, there is a need for high-quality randomized, controlled, and double-blinded trials with adequate designs and significance. Further investigation is necessary in order to understand the mechanism of LLLT effects on diabetic ulcers.

## Figures and Tables

**Table 1:** Compilation of the Wagner and the Armstrong Ulcer Grade Classification System [5, 6].

	Wagner 0	Wagner 1	Wagner 2	Wagner 3	Wagner 4	Wagner 5
Armstrong A	Pre- or postulcerative lesions	Superficial ulcer	Penetration to tendon, joint capsule	Penetration to bone, joint	Gangrene of digit	Gangrene of foot requiring disarticulation
Armstrong B	With infection	With infection	With infection	With infection	With infection	With infection
Armstrong C	With ischemia	With ischemia	With ischemia	With ischemia	With ischemia	With ischemia
Armstrong D	With ischemia and infection	With ischemia and infection	With ischemia and infection	With ischemia and infection	With ischemia and infection	With ischemia and infection

**Table 2:** Clinical trials on leg ulcer treatment with LLLT in diabetic ulcers.

Study	Study design	Participants	Intervention	Outcome measures	Treatment outcome
Schindl et al., 1998 [13]	Randomized, double-blinded, placebo- controlled trial	30 diabetic subjects with foot ulcer/gangrene	Single treatment HeNe laser 632.8 nm, 30 J/cm^2^ , 50 min	Temperature	Increase of microcirculation due to athermic laser irradiation

Schindl et al., 1999 [14]	Case studies	20 patients with diabetes (*n* = 8), arterial insufficiency (*n* = 5), radio damage (*n* = 4), and autoimmune vasculitis (*n* = 3)	30 mW helium neon laser 632.8 nm, 30 J/cm^2^, 3 times weekly, 16–24 weeks until wound closure	Necessary treatments until wound closure	Ulcers due to radio damage healed significantly faster than those caused by diabetes autoimmune vasculitis and required lower time than that in radiodermatitis (nonsignificant);wound size was a predicting parameter but not wound depth and prior treatment

Saltmarche, 2008 [15]	Prospective comparative clinical trial	21 open wounds and 4 “at risk” closed areas, and chronic (> or =3 months) and acute (<3 months) ulcers due to pressure, venous insufficiency, and diabetes	5 times per week in first week, 3 times a week for more 8 weeks, 785 nm	Reduction of size	61.9% of open wounds had significant reduction of size, 42.8% had 100% closure, 14.3% had some improvement, and 23.8% had no change no significant difference between chronic and acute wounds

Zhou et al., 2008 [16]	Randomized, molecular-biological analysis	60 patients with 84 chronic dermal ulcers in diabetes patients and others	Daily till healing Helium neon (HeNe) laser, 632.8 nm	Healing rate, immunohistochemical analysis: HSP70, HSF1	Expression of HSF1, HSP70, and RNA levels of HSF1 and HSP70 in laser group was significantly higher

Minatel et al., 2009 [17]	Randomized, placebo-controlled, double-blinded trial	14 patients with 23 chronic diabetic ulcers	Twice a week till healing, at most 90 days, 660 nm and 890 nm, 3 J/cm^2^, 30 sec/5 cm^2^	Healing rate and granulation	Treatment group had more granulation (day 30: 56%) and faster healing (day 30: 79.2%), 58.3% healed fully (1 ulcer placebo group); 75% ulcer healed 90–100% day 90 (one ulcer placebo group)

Kaviani et al., 2011 [18]	Randomized, placebo-controlled, double-blinded trial	23 patients, diabetic foot wounds for at least three months	2 times a week for 2 weeks then every second day till healing, 685 nm, 10 J/cm^2^ 200 sec	Reduction of ulcer size and healing time	Significant reduction of ulcer size of 58 ± 10.4% in laser group to 23.5 ± 14.1% two weeks after treatment; healing time of treatment group: 11 weeks, placebo: 14 weeks, but not significant

Kajagar et al., 2012 [19]	Randomized controlled trial	68 patients with chronic diabetic foot ulcers, conventional care + LLLT (*n* = 34) versus conventional care alone	Daily treatment for 15 days, 2–4 J/cm^2^ Power, 60 mW Frequency, 5 kHz	Ulcer size	Significant reduction of percentage of ulcer area LLLT group

Landau et al., 2011 [20]	Randomized, placebo-controlled, double-blinded trial	14 patients with diabetic ulcers, 2 patients with venous ulcers	Twice daily for 12 weeks broadband (400–800 nm) 43.2 J/cm^2^, 4 minutes	Healing rate Reduction of size Wound closure time	Treatment group Healing: 90%, reduction of size: 89%, mean/median Wound closure time: 7.14/11.16 weeks Placebo group Healing: 33%, reduction of size: 54%, mean/median Wound closure time: 11.5 weeks

## Abbreviations

ATP: Adenosine triphosphate
CNKI: China National Knowledge Infrastructure
DFI: Diabetic foot infection
DFS: Diabetic foot syndrome
GaALAs-laser: Gallium-aluminum-arsenide laser
GaAs-laser: Gallium-arsenide laser
HSF1: Heat shock factor 1
HSP70: Heat shock protein 70
LED: Light-emitting diode
LILI: Low-intensity laser irradiation
LLLT: Low level laser therapy
LLNB: Low Level Narrow Band (Light)
MMP: Matrix metalloproteinase
mRNA: Messenger ribonucleic acid
NO: Nitric oxide
PCR: Polymerase chain reaction
ROS: Reactive oxygen species
RT-PCR: Reverse transcription polymerase chain reaction
TCM: Traditional Chinese Medicine
TGF: Transforming growth factor.
